# Complete genome sequence of *Picosynechococcus* sp. strain NKBG15041—a fast-growing marine *Cyanobacterium*

**DOI:** 10.1128/mra.00560-25

**Published:** 2025-07-31

**Authors:** Akiyo Yamada, Takahiro Ogawa, Tomoko Yoshino, Tsuyoshi Tanaka, Masafumi Yohda

**Affiliations:** 1 Department of Biotechnology and Life Science, Tokyo University of Agriculture and Technology 13125, Tokyo, Japan; Montana State University, Bozeman, Montana, USA

**Keywords:** *Cyanobacterium*, stress

## Abstract

Here, we report the complete genome sequence of *Picosynechococcus* sp. strain NKBG15041c, which was isolated from coastal seawater in Okinawa Prefecture, Japan. In addition, we determined the complete sequences of three associated plasmids. This genome information provides important insights into the mechanism of understanding the high stress tolerance of this strain.

## ANNOUNCEMENT

*Picosynechococcus* sp. strain NKBG15041c, previously named *Synechococcus* sp. strain NKBG15041c, was isolated from sand containing seawater obtained from a sandy beach in Okinawa Prefecture, Japan. The strain was enriched by culture under light irradiation using BG11 medium adjusted to 3% NaCl and pH 7.4 and was selected as a fast-growing clone on a BG11 agar plate (1). This strain exhibits greater resistance to thermal and acidic stress than other *Cyanobacteria*, such as *Synechocystis* sp. PCC 6803. Notably, P. NKBG15041c was able to grow in medium at pH 4 for 4 days, whereas S. PCC 6803 failed to grow under such acidic conditions. Its draft genome sequence was previously reported (2).

P. NKBG15041c was cultivated in BG11 medium supplemented with 3% NaCl under continuous illumination (50  µmol photons m⁻² s⁻¹) at 30°C with shaking at 100  rpm. Cells were grown until the OD₆₆₀ reached 0.4 and then harvested by centrifugation at 8,000 × g for 10 min at 4℃. Genomic DNA was extracted from bacterial pellets using ISOIL for Bead Beating (NIPPON GENE) according to the manufacturer’s instructions. Following RNase treatment for 30 min at 4°C, the DNA was purified by phenol-chloroform extraction and ethanol precipitation.

For Illumina sequencing, 500  ng of genomic DNA was fragmented to a target size of 350  bp (range: 250–650  bp) using a Covaris M220 Focused-ultrasonicator and purified using PCR Clean DX Beads (Aline Biosciences). Library preparation was performed using the NEB Next Ultra II DNA Library Prep Kit for Illumina (NEB). The resulting sequencing libraries were quantified using the Qubit DNA Assay (Thermo Fisher Scientific), and their fragment size distribution was assessed using the TapeStation D1000 ScreenTape (Agilent). The library was sequenced with 150 bp pair-end reads on the NovaSeq 6000 Sequencing System. For PacBio sequencing, 3 µg of genomic DNA was sheared to an appropriate fragment length by g-TUBE (Covaris) and used for library construction with the SMRTbellprep kit 3.0 (PacBio). The library concentration was quantified using a Qubit 3.0 Fluorometer (Invitrogen), and its size distribution was assessed using an Agilent 2100 Bioanalyzer system. Subsequent steps were carried out according to the manufacturer’s instructions. The library was sequenced using HiFi chemistry on the PacBio Sequel IIe platform. A summary of sequence data is shown in the table.

Genome assembly, integrating PacBio long reads and Illumina short reads, was performed using B-assembler (3). Circularity of the resulting six contigs was assessed using Circlator (4), confirming that four were circular. The longest circular contig represents the complete genome of P. NKBG15041c, comprising 2,845,435  bp with a G + C content of 49.8%. Genome annotation was conducted using DFAST version 1.6.0 (5), identifying 2,645 coding sequences, six rRNA genes, and 42 tRNA genes (Table 1). Default parameters were used except where otherwise noted.

**TABLE 1:** Summary of sequence data and features of genome and plasmids of *Picosynechococcus* sp. strain NKBG15041c

Summary of sequence data						
**PacBio**						
**Reads**	**Bases**	**Min**	**Max**	**Average**	**N50**	
120,238	735,864,165	567	22,805	6,120.06	7,042	
**Illumina**						
**Reads**	**Length**	**Yields**	**Q20 (%**)	**Q30 (%**)	**Q40 (%**)	
7,588,634	150	1,138,295,100	97.66	93.4	50.35	
**Features of genome and plasmids**						
	**Length**	**Coverage**	**GC Content (%**)	**CDSs**	**rRNA**	**mRNA**
Genome	2,845,435	20 x	49.8	2,645	6	42
Plasmid 1	122,908	23 x	46.3	92	0	0
Plasmid 2	109,665	19 x	44	78	0	0
Plasmid 3	68,608	23 x	45.1	67	0	0

Average Nucleotide Identity (ANI) analysis, performed using JSpeciesWS version 4.1.1 (6), revealed that P. NKBG15041c shares 98.53% ANI with *Synechococcus* sp. NIES-970 (AP017959) (7), while the ANI with S. PCC 6803 (BA000022)(8) was 68.3%. The remaining three circular contigs showed high homology with three plasmids from S. NIES-970 (AP017960–AP017962).

## Data Availability

The whole-genome sequences have been deposited in GenBank/DDBJ/ENA under accession numbers AP040363 (bacterial genome assembly), AP040364, AP040365, and AP040366 (Plasmid sequences). Raw sequence data are available in Dryad Digital Repository, https://doi.org/10.5061/dryad.wdbrv161k. The isolates have been deposited under BioSample accession number SAMD00911255 and BioProject accession number PRJDB1488.
